# Supplementary material for: Neuronal CaMKK2 promotes immunosuppression and checkpoint blockade resistance in glioblastoma
Source: Nat Commun. 2022 Oct 29;13:6483. doi: 10.1038/s41467-022-34175-y (PMC9617949; doi:10.1038/s41467-022-34175-y)
Supplement: Supplementary file 1 — Supplementary Information [file 41467_2022_34175_MOESM1_ESM.pdf]

Supplementary Figure 1

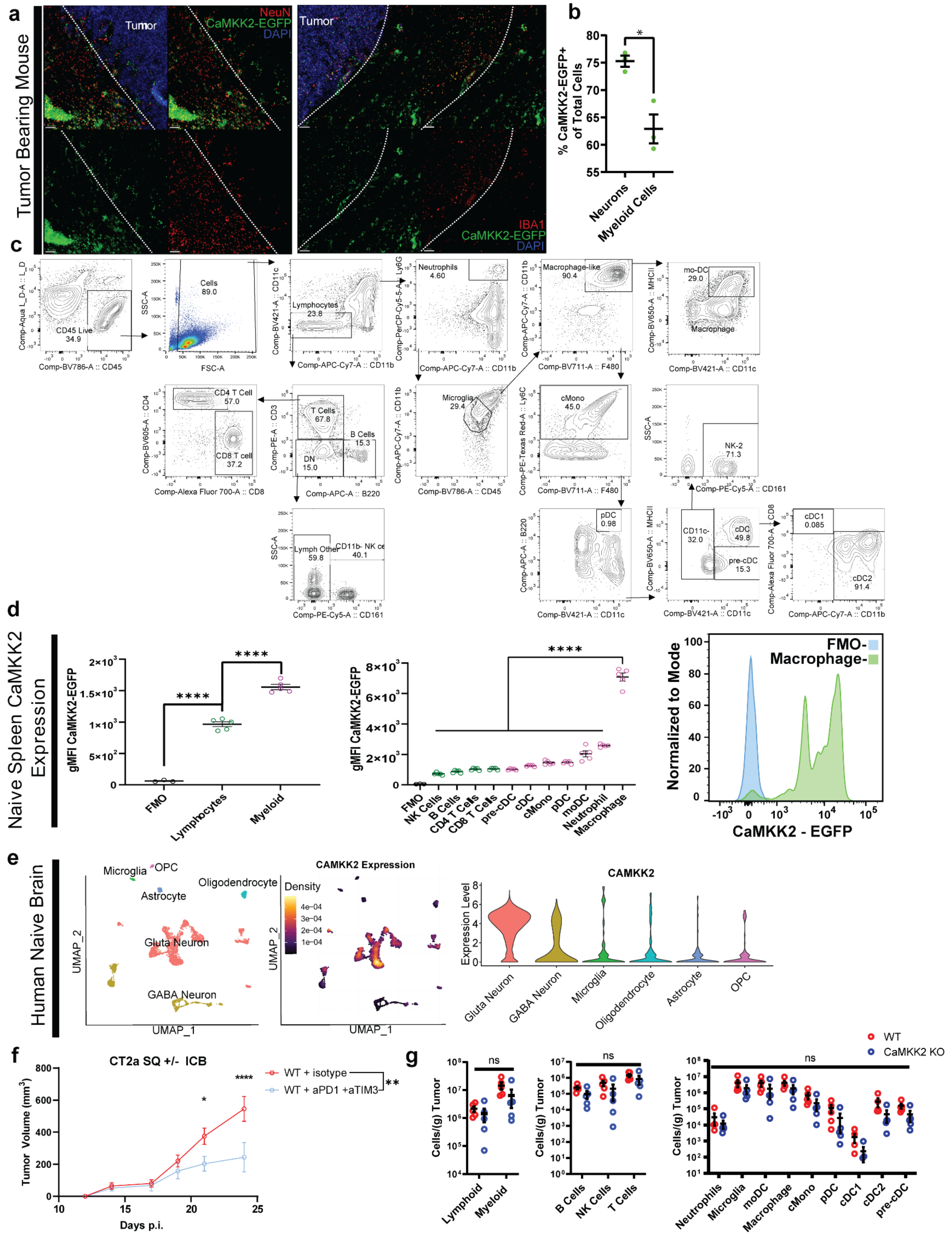

Supplementary Figure 1**. CaMKK2 is highly expressed in neurons and myeloid cells in humans and mice. a,b** Confocal images of D14 brain tumor sections of CaMKK2-EGFP reporter mice. NeuN and IBA1 were used to identify neurons and myeloid cells respectively. Imaris was used to determine the colocalization of either Iba1 or NeuN and CaMKK2-EGFP signal. Y-axis denotes % of total cells of that type that are EGFP+. N = 3 mice per staining panel, two-tailed t-test.* = p = .0119. Scale bar represents 70µm. **c** Flow cytometry gating strategy that was utilized in Fig.1a and Supplementary Fig.1d,g **d** Spleens were harvested from naïve WT (FMO) or transgenic CaMKK2-EGFP reporter mice and stained with a multi-color flow panel to resolve major immune populations. N = 3 FMO, and n = 5 per other groups, ANOVA p < .05 with post-hoc unadjusted Fisher LSD two-tailed t-test. **** = p < .0001. **e** UMAP plot of Naïve Human Brain cells, and density and violin plots showing CaMKK2 expression within those cell types. Data pulled from Allen Institute for Brain Science (https://portal.brain-map.org/atlases-and-data/rnaseq/human-m1-10x) **f** 250k CT2a was implanted in the flank of WT mice and tumor growth was monitored. Either 400ug isotype or a combination therapy of 200ug aPD1 and 200ug aTIM3 was administered on D3 p.i and every 3 days through D15 for a total of 5 treatments. n = 8 per group, 2-way ANOVA p<.05 with post-hoc unadjusted Fisher LSD two-tailed t-test.** = p = .0097, **** = adj. p < .0001. **g** Tumor-bearing hemispheres were harvested from WT or CaMKK2 KO mice on D14 and stained with a multi-color flow panel to resolve major immune populations. n = 5, Two-way RM ANOVA p< .05 with post-hoc two-tailed unadjusted Fisher LSD t-test. **b,d,f,g** Data are presented as mean ± S.E.M. Source data are provided as a Source Data file.

Supplementary Figure 2

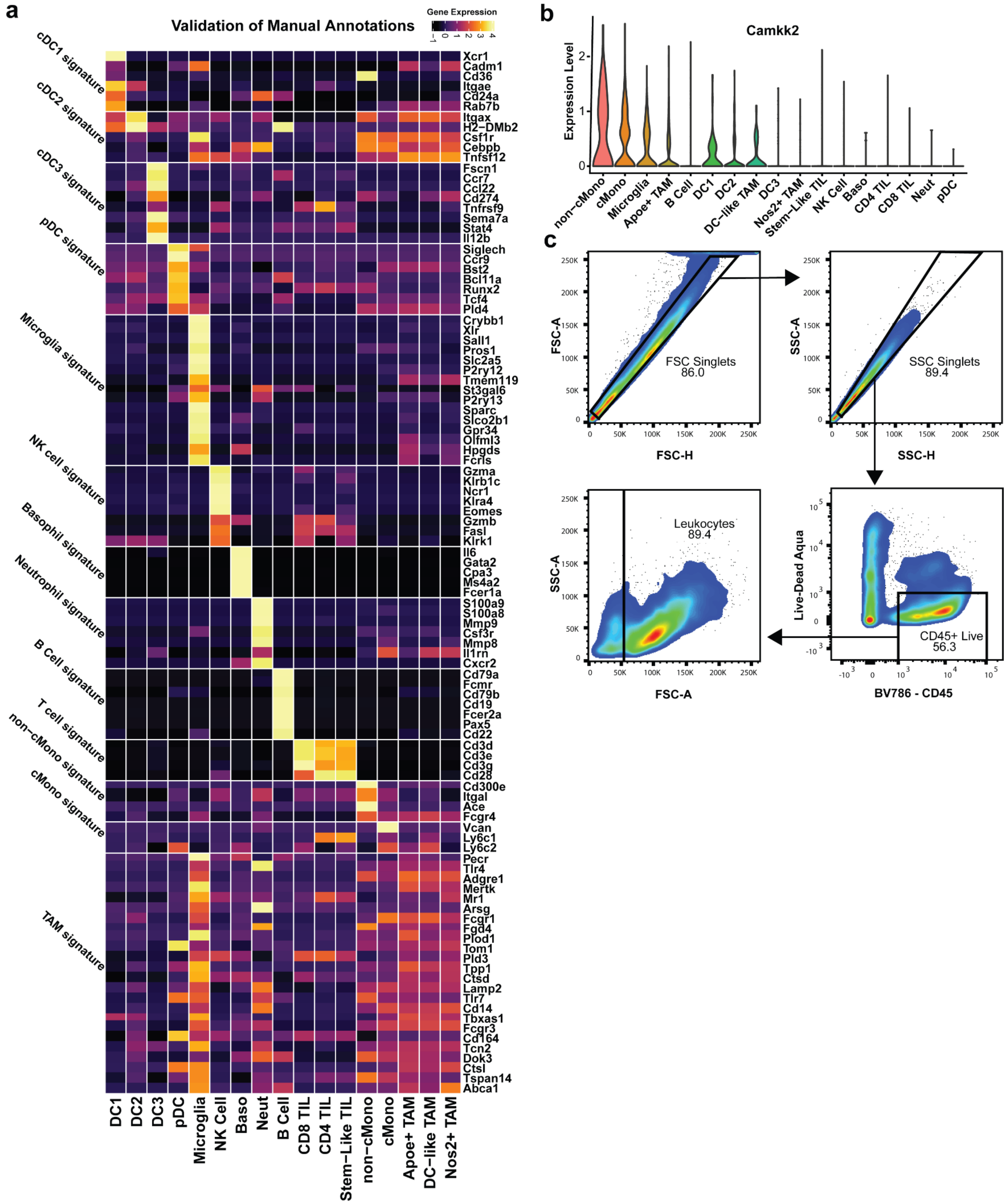

Supplementary Figure 2. **scRNA-seq clusters highly express previously identified cell signatures. a** Scaled expression of immune signature genes in the cell clusters identified in the scRNA-seq dataset. **b** Violin plots of CaMKK2 expression levels across immune subsets of WT cells. **c** Cell sorting strategy used for scRNA-seq experiment.

Supplementary Figure 3

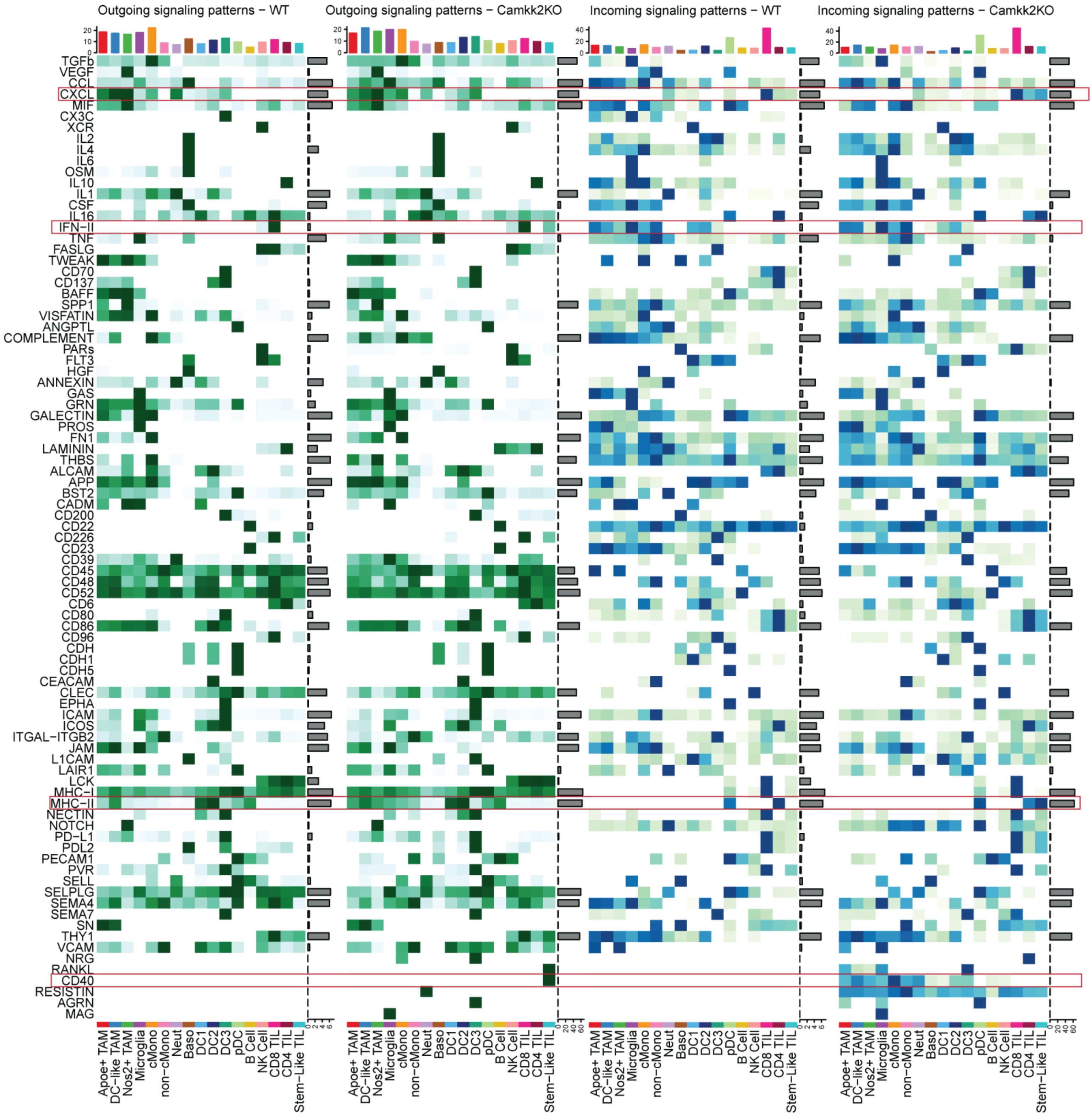

Supplementary Figure 3**. CXCL, IFN-II, MHC-II, and CD40 interaction pathways are enriched in myeloid cells of the CaMKK2 deficient tumor immune microenvironment.** Relative overall interaction strength for the observed receptor-ligand interaction pathways is indicated in this heatmap for the cell clusters identified in the scRNA-seq data. Bar plots on the perimeter of the heatmaps represent the sum of signaling strengths for that pathway across cell types (right side) or the sum of all pathway interaction strengths for a given cell type (on the top). Outgoing signaling patterns refer to cells which express the ligand for that pathway and are considered the “sender”. Incoming signaling patterns refer to cells which express the receptor for that pathway and are considered the “targets”.

Supplementary Figure 4

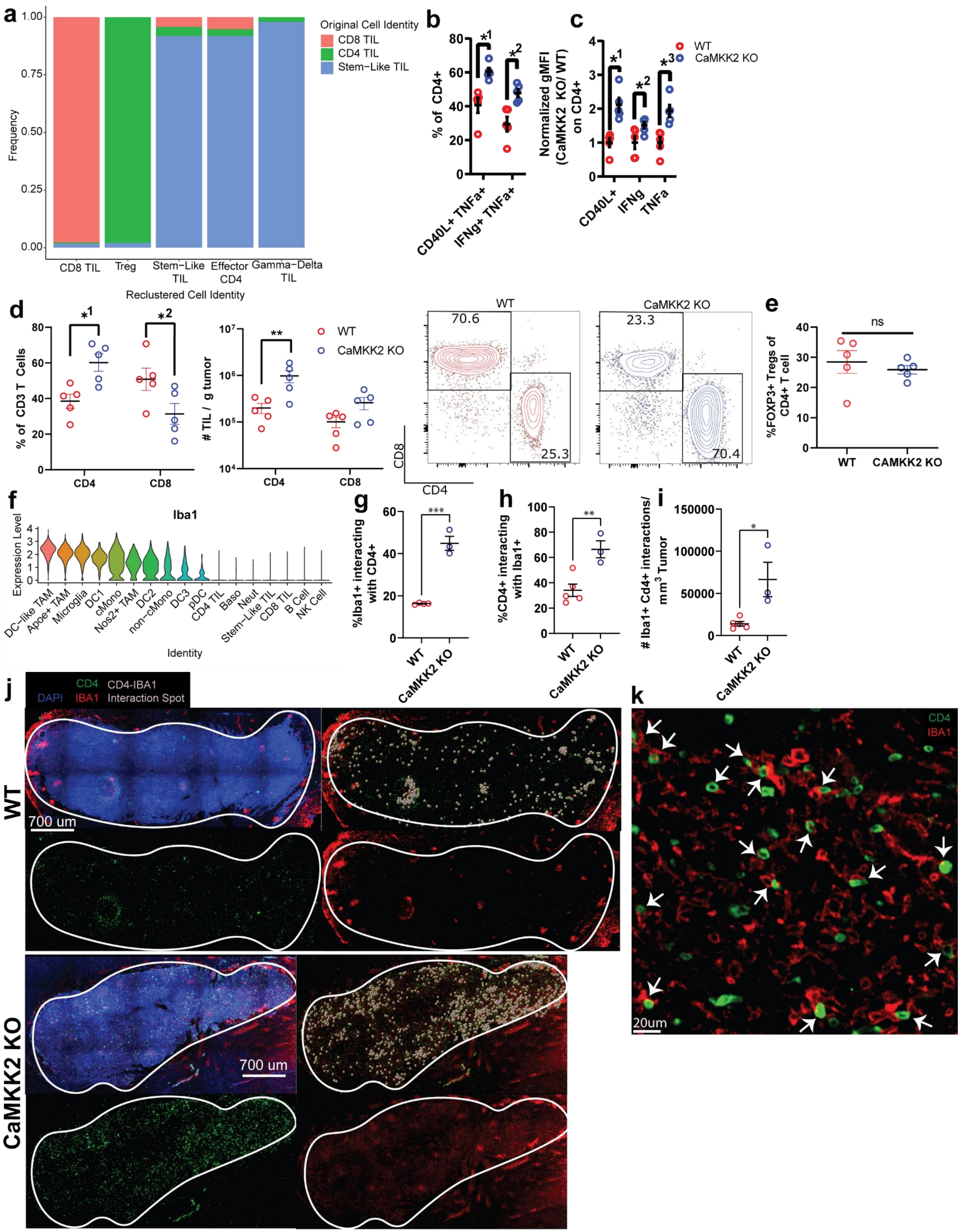

Supplementary Figure 4. **CaMKK2 reduces CD4-myeloid interactions in the glioma tumor microenvironment. a** Stacked bar plot showing the frequency of the originally identified TIL clusters (Fig. 2b) in the new clusters identified through reclustering (Fig.3a).**b-e** Tumor bearing-hemispheres were harvested on D14 post CT2a implantation and assessed by flow cytometry. **b,c** n = 5 per genotype, two-tailed Holm-Sidak adjusted t-test. **b** $*^1$ = adj. p = .007, $*^2$ = adj. p = .007. **c** Each sample replicate was normalized to the average WT gMFI, $*^1$ = adj. p = .005, $*^2$ = adj. p = .045, $*^3$ = adj. p = .008. **d** n = 5 per genotype, two-way RM ANOVA p < .05 with post-hoc unadjusted two-tail Fisher LSD t-test, $*^1$ = p = .011, $*^2$ = p = .020, ** = p = .002. **e** n = 5 per genotype, two-tailed t-test. **f** Iba1 expression levels in cell types identified in scRNA-seq. **g** Percentage of Iba1+ cells which co-localize with CD4+ cells found by microscopy images of D14 tumor-bearing hemispheres. n = 5 WT, n = 3 CaMKK2 KO, two-tailed t-test, *** = p = .0002. **h** Percentage of CD4+ cells which co-localize with Iba1+ myeloid cells found by microscopy images of D14 tumor-bearing hemispheres. n = 5 WT, n = 3 CaMKK2 KO, two-tailed t-test, ** = p = .0073. **i** Number of Iba1+ myeloid cells which co-localize with CD4+ cells found per mm^3 tumor by microscopy images of D14 tumor-bearing hemispheres. n = 5 WT, n = 3 CaMKK2 KO, two-tailed t-test, * = p = .0143. **j** Representative images of D14 tumor-bearing hemispheres stained with CD4, Iba1, and DAPI to identify CD4+ cells, myeloid cells, and nuclei respectively. Spot surfaces were created around cells and interaction was defined as co-localization of Iba+ and CD4+ spots. **k** Representative image of a single z-plane within the tumor microenvironment showing CD4+ Iba1+ interactions. **b-e, g-i** Data are presented as mean ± S.E.M. **j-k** Experiments were independently repeated twice. Source data are provided as a Source Data file.

Supplementary Figure 5

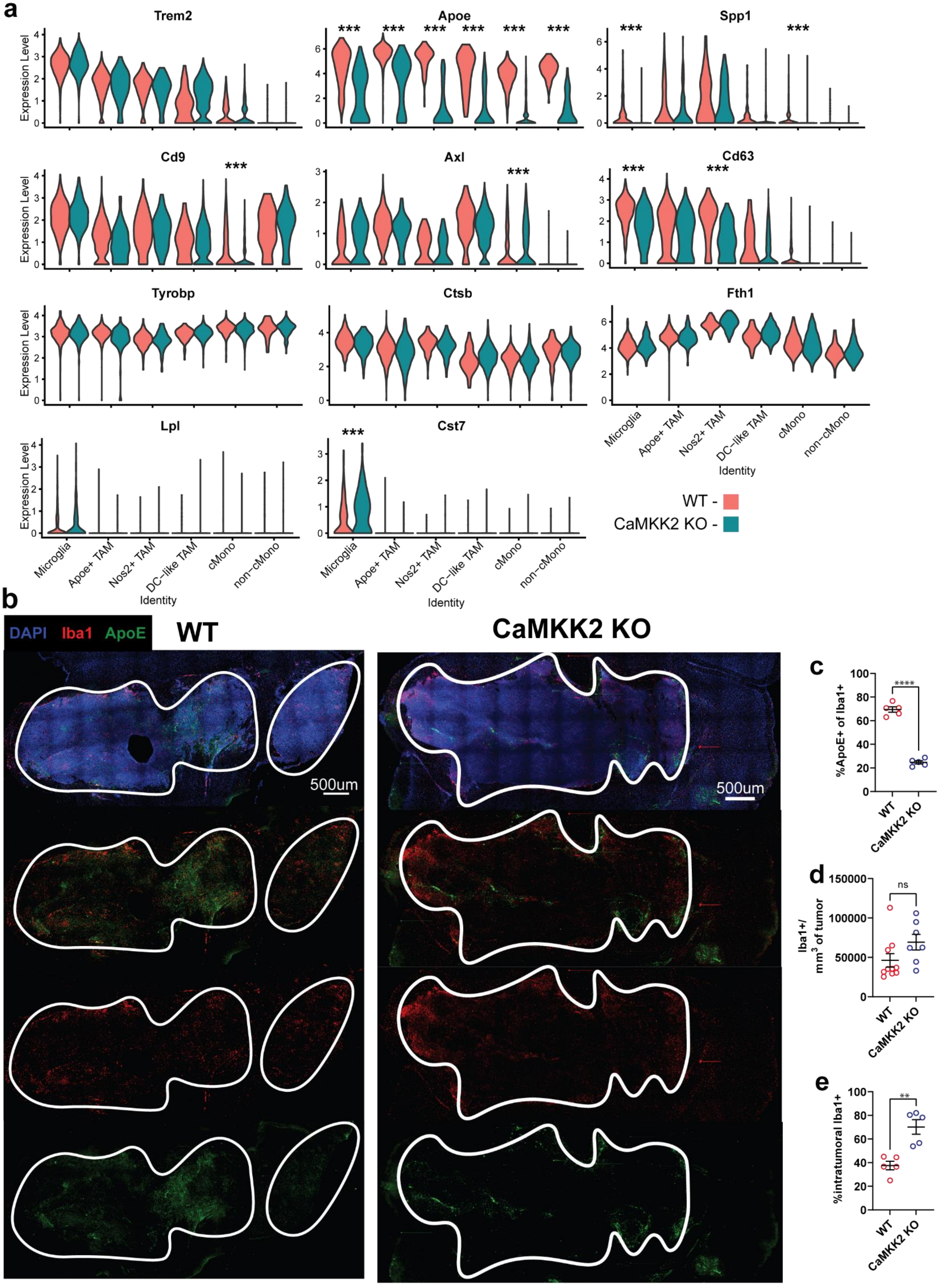

Supplementary Figure 5. **CaMKK2 promotes Apoe^{+} phenotype and restricts intratumoral infiltration of myeloid cells. a** Violin plots of DAM signature genes stratified by genotype and cell type. MAST was used for differential expression testing. *** = adj. $p < .005$. **b** Representative images of D14 tumor-bearing hemispheres stained with Iba1 to identify myeloid cells, and Apoe to identify Apoe+ TAMs. Spot surfaces were created around Iba+ cells and %ApoE+ refers to Apoe signal co-localizing with Iba1+ spots. Experiment was independently repeated twice. **c** Percent of Iba1+ cells which colocalized with Apoe signal in confocal microscopy images of D14 tumor-bearing hemispheres. n = 5 per genotype, two-tailed t-test. **** = $p < .0001$ **d** Number of Iba1+ cells found per mm^3 tumor by confocal microscopy images of D14 tumor-bearing hemispheres. n = 10 WT, n = 7 CaMKK2 KO, two-tailed t-test. Results are combined from two independent experiments **e** Percentage of intratumoral Iba1+ cells of total Iba1+ cells identified, in D14 tumor-bearing hemispheres, by confocal microscopy. n = 5 per genotype, two-tailed t-test. ** = p = .0018 **c-e** Data are presented as mean ± S.E.M. Source data are provided as a Source Data file.

Supplementary Figure 6

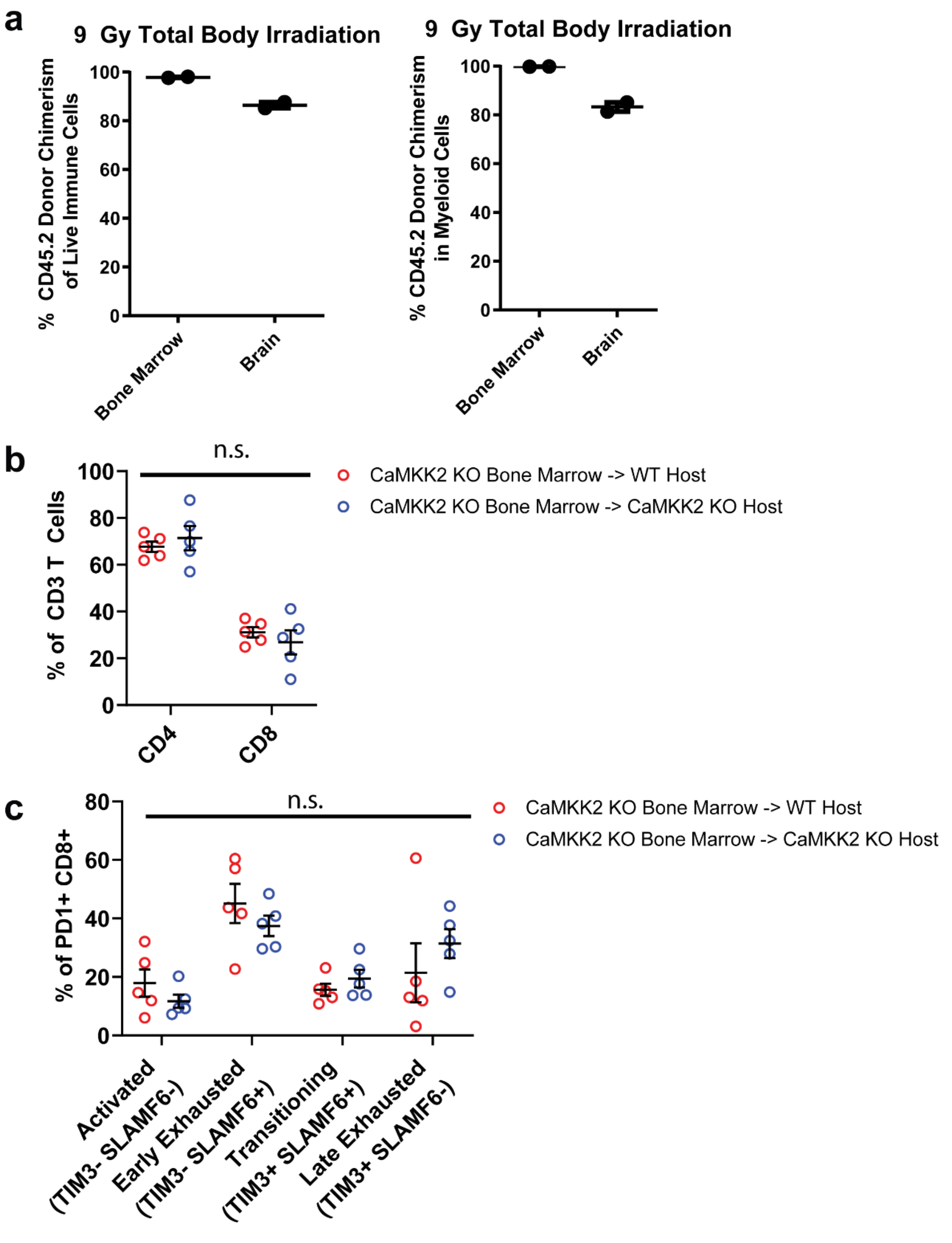

Supplementary Figure 6. **Hematopoietic CaMKK2 deficiency is sufficient to phenocopy total body CaMKK2 deficiency T cell phenotypes. a** CD45.1 Naïve mice were irradiated with 9 Gy irradiation and then received CD45.2 bone marrow. After recovering for 8 weeks donor chimerism was assessed in the bone marrow and brain. n = 2 mice. **b,c** Either WT or CaMKK2 KO mice received bone marrow from CaMKK2 KO mice after receiving 9Gy radiation. Recipient mice recovered for 8 weeks prior to tumor implantation of CT2a. Tumor-bearing hemispheres were harvested on D14 post tumor implantation and stained with a multi-color flow panel to assess TIL abundance and exhaustion. n = 5 per group, two-way RM ANOVA $p > .05$. **a-c** Data are presented as mean ± S.E.M. Source data are provided as a Source Data file.

Supplementary Table 1

Antigen	Company	Clone	Fluorophore	Catalog Number	Dilution	Panel
Trem2	R&D Systems	237920	PE	FAB17291P	1:50	TAMs
Tim3	invitrogen	RMT3-23	PE	12-5870-82	1:200	Exhaustion
CD154/CD40L	BioLegend	MR1	PE	106506	1:200	Effector CD4
Ly-6C	BioLegend	HK1.4	PE/Dazzle	128043	1:200	Broad Immunophenotyping
IFN-gamma	BioLegend	XMG1.2	PE/Dazzle	505846	1:200	Effector CD4
CD44	BioLegend	IM7	PE-Cyanine7	103029	1:400	Effector to Treg
Cd40	Invitrogen	1C10	APC	17-0401-82	1:100	TAMs
Foxp3	invitrogen	FJK-16s	APC	17-5773-80	1:100	Effector to Treg, Effector CD4
TOX	invitrogen	TXRX10	eF660	50-6502-82	1:200	Effector CD4
SLAMF6	invitrogen	eBio13G3-19D	APC	17-1508-82	1:200	Exhaustion
CD4	BioLegend	RM4-5	AF700	100536	1:200	Effector CD4, Exhaustion
CD8a	BioLegend	53-6.7	APC/Cy7	100714	1:100	Effector to Treg, Exhaustion
CD8a	Biolegend	53-6.7	Brilliant Violet 421	100753	1:200	Effector CD4
CD3	BD Biosciences	17A2	Brilliant Violet 421	564008	1:200	Exhaustion
CD62L	BioLegend	MEL-14	Brilliant Violet 421	104435	1:100	Effector to Treg
CD45	invitrogen	30-F11	evolve 605	83-0451-42	1:100	Effector to Treg, Effector CD4, Exhaustion
MRC1/CD206	Biolegend	C068C2	Brilliant Violet 605	141721	1:100	TAMs
CD3e	BD Biosciences	145-2C11	Brilliant Violet 650	564378	1:100	Effector to Treg
CD4	BD Biosciences	GK1.5	Brilliant Violet 786	563331	1:100	Effector to Treg
TNF-alpha	BioLegend	MP6-XT22	Brilliant Violet 785	506341	1:200	Effector CD4
CD 279/PD-1	BD Biosciences	J43	Brilliant Violet 786	744548	1:200	Exhaustion
CD11c	BioLegend	N418	Brilliant Violet 421	117343	1:200	Broad Immunophenotyping
Zombie Aqua	BioLegend	N/A	Brilliant Violet 510	423102	1:400	Broad Immunophenotyping, Effector to Treg, Effector CD4, Exhaustion
CD4	eBioscience	RM4-5	Brilliant Violet 605	83-0042-42	1:400	Broad Immunophenotyping
MHC II	eBioscience	M5/114.15.2	Brilliant Violet 650	64-5321-82	1:400	Broad Immunophenotyping
F4/80	BioLegend	BM8	Brilliant Violet 711	123417	1:200	Broad Immunophenotyping

CD45.2	BD Biosciences	104	Brilliant Violet 786	563686	1:100	Broad Immunophenotyping
Ly6G	BD Biosciences	1A8	PerCP-Cy5.5	566435	1:100	Broad Immunophenotyping
CD3	eBioscience	143-2C11	PE	12-0031-82	1:100	Broad Immunophenotyping
NK1.1	BioLegend	PK136	PE-Cy5	108702	1:100	Broad Immunophenotyping
CD43	BD Biosciences	S7	PE-Cy7	562866	1:100	Broad Immunophenotyping
B220	BioLegend	RA3-6B2	APC	103212	1:200	Broad Immunophenotyping
CD8	eBioscience	53-6.7	AF700	56-0081-82	1:200	Broad Immunophenotyping
CD11b	BD Biosciences	M1/70	APC-Cy7	557657	1:400	Broad Immunophenotyping

Supplementary Table 1. **Flow cytometry antibodies list.** This table provides the details for the flow cytometry antibodies used within this manuscript.

Supplementary Table 2

Antigen	Company	Clone	Host	Target	Fluorophore	Catalog Number	Dilution
CD4	abcam	EPR19514	Rabbit	Mouse	N/A	ab183685	1 to 500
Rabbit Fc	invitrogen	Polyclonal	Donkey	Rabbit	AF647	A31573	1 to 500
GFP	ThermoFisher	Polyclonal	Chicken	Mouse	N/A	A10262	1 to 500
Chicken Fc	Jackson ImmunoResearch	Polyclonal	Donkey	Chicken	AF488	AB_2340375	1 to 500
NeuN	abcam	EPR12763	Rabbit	Mouse	N/A	ab177487	1 to 500
Goat Fc	invitrogen	Polyclonal	Donkey	Goat	AF568	A11057	1 to 500
Iba1	abcam	EPR16588	Rabbit	Mouse	N/A	ab178846	1 to 500
ApoE	abcam	EPR19392	Rabbit	Mouse	N/A	ab183597	1 to 200
Iba1	abcam	Polyclonal	Goat	Mouse	N/A	ab5076	1 to 500

Supplementary Table 2. **Immunofluorescence antibodies list.** This table provides the details of the immunofluorescence antibodies used within this manuscript.
