# Supplementary material for: Neuronal CaMKK2 promotes immunosuppression and checkpoint blockade resistance in glioblastoma
Source: Nat Commun. 2022 Oct 29;13:6483. doi: 10.1038/s41467-022-34175-y (PMC9617949; doi:10.1038/s41467-022-34175-y)
Supplement: Supplementary file 3 — Description of Additional Supplementary Files [file 41467_2022_34175_MOESM3_ESM.pdf]

Supplementary Data 1. **Conserved markers between genotypes for cell clusters in Fig. 2b.** Highly expressed genes that were conserved between genotypes for each cell cluster identified in Fig. 2b. MAST was used for differential expressing testing, which supplies adjusted p-values.

Supplementary Data 2. **Differentially expressed genes between genotypes for cell clusters in Fig. 2b.** Genes that were differentially expressed between genotypes for each cluster identified in Fig. 2b. MAST was used for differential expressing testing, which supplies adjusted p-values.

Supplementary Data 3. **Conserved Markers Between Genotypes for Cell Clusters in Fig. 3a.** Highly expressed genes that were conserved between genotypes for each cell cluster identified in Fig. 3a. MAST was used for differential expressing testing, which supplies adjusted p-values.

Supplementary Data 4. **Differentially expressed genes between genotypes for cell clusters in Fig. 3a.** Genes that were differentially expressed between genotypes for each cluster identified in Fig. 3a. MAST was used for differential expressing testing, which supplies adjusted p-values.

Supplementary Data 5. **Differentially expressed genes between DC-like and Apoe TAMs in Fig. 6a.** Genes that were differentially expressed between DC-like and Apoe+ TAMs visualized in Fig. 6a. MAST was used for differential expressing testing, which supplies adjusted p-values.

Supplementary Data 6. **Full enriched GO term list Fig. 6b.** An unabbreviated list of the enriched GO terms visualized in Fig. 6b. Benjamini p-value corrections were used for significance testing.

Supplementary Data 7. **Gene signatures used.** Lists of the various gene signatures used throughout the manuscript.

Supplementary Data 8. **Survival statistics.** This Spreadsheet contains the median survival and 95% CI interval for all groups for each presented survival curve. Statistics were calculated using SAS PROC LIFETEST.
