# Supplementary material for: Neuronal CaMKK2 promotes immunosuppression and checkpoint blockade resistance in glioblastoma
Source: Nat Commun. 2022 Oct 29;13:6483. doi: 10.1038/s41467-022-34175-y (PMC9617949; doi:10.1038/s41467-022-34175-y)
Supplement: Supplementary file 12 — Reporting Summary [file 41467_2022_34175_MOESM12_ESM.pdf]

nature portfolio

Corresponding author(s): William Tomaszewski

Last updated by author(s): Sep 16, 2022

Reporting Summary

Nature Portfolio wishes to improve the reproducibility of the work that we publish. This form provides structure for consistency and transparency in reporting. For further information on Nature Portfolio policies, see our Editorial Policies and the Editorial Policy Checklist.

Statistics

For all statistical analyses, confirm that the following items are present in the figure legend, table legend, main text, or Methods section.

n/a	Confirmed	
☐	☒	The exact sample size (*n*) for each experimental group/condition, given as a discrete number and unit of measurement
☐	☒	A statement on whether measurements were taken from distinct samples or whether the same sample was measured repeatedly
☐	☒	The statistical test(s) used AND whether they are one- or two-sided<br>*Only common tests should be described solely by name; describe more complex techniques in the Methods section.*
☐	☒	A description of all covariates tested
☐	☒	A description of any assumptions or corrections, such as tests of normality and adjustment for multiple comparisons
☐	☒	A full description of the statistical parameters including central tendency (e.g. means) or other basic estimates (e.g. regression coefficient) AND variation (e.g. standard deviation) or associated estimates of uncertainty (e.g. confidence intervals)
☐	☒	For null hypothesis testing, the test statistic (e.g. *F*, *t*, *r*) with confidence intervals, effect sizes, degrees of freedom and *P* value noted<br>*Give P values as exact values whenever suitable.*
☒	☐	For Bayesian analysis, information on the choice of priors and Markov chain Monte Carlo settings
☒	☐	For hierarchical and complex designs, identification of the appropriate level for tests and full reporting of outcomes
☒	☐	Estimates of effect sizes (e.g. Cohen's *d*, Pearson's *r*), indicating how they were calculated

Our web collection on statistics for biologists contains articles on many of the points above.

Software and code

Policy information about availability of computer code

Data collection	Flow Cytometry data was collected using FACS Diva software v.9 (BD Biosciences)
Data analysis	The R code that was used to perform the scRNA-seq analysis can be found on Github: https://github.com/wht10/CT2A_scRNAseq_CaMKK2KOvWT.<br><br>Flow cytometry data was analyzed using FlowJo v.10 (Tree Star)<br><br>FASTQ files were demultiplexed from the raw sequencing reads (bcl2fastq, v2.20), aligned to the mouse mm10 reference genome (cellranger, v4.0.0), and raw gene count matrices were generated using STAR (v2.7.5c).<br><br>Downstream analysis was performed using the R software Seurat package (v4.0.3)<br><br>Nebulosa (v.1.0.2) was used to create density plots.<br><br>Cell-cell communication analysis was performed using the CellChat 57 (v.1.0.0) software package in R.<br><br>Imaris v.9.6.0 (Oxford Instruments) was used to process and analyze images.<br><br>Statistical tests were completed using GraphPad v.9.2.0 (Prism).

For manuscripts utilizing custom algorithms or software that are central to the research but not yet described in published literature, software must be made available to editors and reviewers. We strongly encourage code deposition in a community repository (e.g. GitHub). See the Nature Portfolio guidelines for submitting code & software for further information.

Data

Policy information about availability of data

All manuscripts must include a data availability statement. This statement should provide the following information, where applicable:

- Accession codes, unique identifiers, or web links for publicly available datasets
- A description of any restrictions on data availability
- For clinical datasets or third party data, please ensure that the statement adheres to our policy

Unprocessed scRNA-seq data has been uploaded to NCBI Gene Expression Omnibus ((https://www.ncbi.nlm.nih.gov/geo/) under data repository accession number https://www.ncbi.nlm.nih.gov/geo/query/acc.cgi?acc=GSE197879. The processed Seurat objects have also been made available through zenodo under record number https://zenodo.org/record/6654420. The data necessary to reproduce the graphs presented within this manuscript are provided in the Source Data file. The publicly available data used in this study were obtained from the GENT2 database http://gent2.appex.kr/gent2/, from the GBMseq portal http://gbmseq.org/, and from the Allen Brain Map https://portal.brain-map.org/atlases-and-data/rnaseq/human-m1-10x. The mm10 reference assembly is available through GenBank under accession code GCA_000001635.2 (https://www.ncbi.nlm.nih.gov/assembly/GCF_000001635.20/). The remaining data are available within the article, Supplementary Information or Source Data file.

Field-specific reporting

Please select the one below that is the best fit for your research. If you are not sure, read the appropriate sections before making your selection.

[x] Life sciences   [ ] Behavioural & social sciences   [ ] Ecological, evolutionary & environmental sciences

For a reference copy of the document with all sections, see nature.com/documents/nr-reporting-summary-flat.pdf

Life sciences study design

All studies must disclose on these points even when the disclosure is negative.

Sample size	Power analysis was not used to predetermine sample size. Sample sizes were instead determined based on historical sample sizes that were capable of detecting biologically significant differences for certain assays. If no historical data was available, pilot experiments were performed to determine the relative variability of the assay.
Data exclusions	No data was excluded.
Replication	All experiments were successfully repeated at least twice.
Randomization	Mice were randomly assigned to treatment groups within a given genotype. All experiments involving treatments were performed in-vivo and not in-vitro. For non-treatment, in-vivo or ex-vivo experiments, mice were randomly selected from age,sex, and genotype matched breeding cohorts.
Blinding	Survival experiments were partially monitored by blinded veterinary staff. Veterinary staff were unable to monitor the experiments everyday so unblinded investigators assisted in survival monitoring.<br><br>Confocal microscopy images were acquired, and analyzed by a blinded co-author.<br><br>All other experiments were analyzed by unblinded investigators due to personnel shortages not allowing for availability of separate investigators for data acquisition and analysis.

Reporting for specific materials, systems and methods

We require information from authors about some types of materials, experimental systems and methods used in many studies. Here, indicate whether each material, system or method listed is relevant to your study. If you are not sure if a list item applies to your research, read the appropriate section before selecting a response.

Materials & experimental systems

n/a	Involved in the study	
[ ]	[x]	Antibodies
[ ]	[x]	Eukaryotic cell lines
[x]	[ ]	Palaeontology and archaeology
[ ]	[x]	Animals and other organisms
[x]	[ ]	Human research participants
[x]	[ ]	Clinical data
[x]	[ ]	Dual use research of concern

Methods

n/a	Involved in the study	
[x]	[ ]	ChIP-seq
[ ]	[x]	Flow cytometry
[x]	[ ]	MRI-based neuroimaging

Antibodies

Antibodies used	Antibodies used are noted in Supplemental Table 8.
Validation	Extensive validation was performed, and detailed in a separate manuscript (PMID: 34597618). Antibodies utilized in this manuscript were optimized by titrating to achieve a maximal staining index.

Eukaryotic cell lines

Policy information about cell lines

Cell line source(s)	C57BL/6 syngeneic CT2a, GL261, and KR158B-Luc were provided by Robert L. Martuza (Massachusetts General Hospital), the National Cancer Institute, and Duane Mitchell (University of Florida), respectively. These cell lines are additionally available commercially (CT2a, Sigma-Aldrich, SCC194; GL261, DSMZ, ACC 802). KR158B-Luc is not currently commercially available.
Authentication	All cell lines were authenticated and tested by IDEXX Laboratories. Interspecies contamination was evaluated by species specific PCR and STR profiling.
Mycoplasma contamination	All cell lines were confirmed to be mycoplasma negative by IDEXX Laboratories.
Commonly misidentified lines (See ICLAC register)	No commonly misidentified cell lines were used in this study.

Animals and other organisms

Policy information about studies involving animals; ARRIVE guidelines recommended for reporting animal research

Laboratory animals	Six- to eight-week-old C57BL/6J, LysMcre, Syn1cre, and CD45.1 mice were purchased from the Jackson Laboratory. CaMKK2-/-, Tg (Camkk2-EGFP)DF129Gsat reporter mice (CaMKK2-EGFP), and CaMKK2fl/fl mice were generously provided by Luigi Racioppi (Duke University). CaMKK2-/-, CaMKK2-EGFP and CaMKK2fl/fl mice have been previously validated. Animals were maintained under pathogen-free conditions at the Cancer Center Isolation Facility of Duke University Medical Center. Experiments were conducted on age and sex matched female mice between 8-12 weeks of age. Animals were maintained under pathogen-free conditions, in temperature and humidity controlled housing, with free access to food and water, under a 12-h light/dark cycle at the Cancer Center Isolation Facility of Duke University Medical Center.
Wild animals	This study did not include wild animals.
Field-collected samples	This study did not include specimens collected from the field.
Ethics oversight	All experimental procedures were approved by the Institutional Animal Care and Use Committee at Duke University Medical Center.

Note that full information on the approval of the study protocol must also be provided in the manuscript.

Flow Cytometry

Plots

Confirm that:

- [x] The axis labels state the marker and fluorochrome used (e.g. CD4-FITC).
- [x] The axis scales are clearly visible. Include numbers along axes only for bottom left plot of group (a 'group' is an analysis of identical markers).
- [x] All plots are contour plots with outliers or pseudocolor plots.
- [x] A numerical value for number of cells or percentage (with statistics) is provided.

Methodology

Sample preparation	Sample preparation is extensively detailed here (PMID: 34597618). IIn brief, tumor-bearing hemispheres were harvested on day 14 post tumor implantation. Tissue was transferred to a Dounce Tissue Homogenizer with 5 mLs of digestion cocktail containing 0.05 mg ml-1 LIberase DL (Roche), 0.05 mg ml-1 Liberase TL (Roche), 0.2 mg ml-1 DNase I (Roche) in HBSS with calcium and magnesium. A cell suspension was obtained after 5-10 strokes with the loose-fitting (A-size) pestle. The cell mixture was then incubated at 37 °C for 15 min in a water bath to obtain a single-cell suspension. The single-cell suspension was then passed through a 70-µm filter. After centrifugation, the cells were resuspended in 1x RBC Lysis Buffer (Thermo Fisher Scientific) for 3 minutes. Myelin was removed from the sample with Percoll centrifugation. Samples were centrifuged and mixed with 30% Percoll (Sigma Aldrich) and centrifuged at 500g for 20 min at 18 °C with no brake. The myelin layer and Percoll were then aspirated and the pellet was re-suspended in PBS before counting on an automated cell counter (Thermo Fisher Scientific).<br><br>For cytokine re-stimulation, samples are resuspended in 1mL RPMI + 10% FBS at 1-2x107 cells ml-1 in a 24 well plate, before viability, extracellular, or intracellular staining. 2 µl Cell Activation Cocktail with Brefeldin A (Biolegend) and 2 µl GolgiStop (BD

Biosciences) were added and then samples were incubated for 4 hours at 37 °C.
If samples were not stimulated, cells were then resuspended at 1-2x107 ml-1 in 100 µl PBS and transferred to a 96-well plate. Before further staining, samples were re-suspended in Zombie Aqua Viability Dye (1:400, Biolegend) and incubated for 30 minutes on ice.

For extracellular staining, samples were incubated with a blocking solution containing 2% Normal Rat Serum (Thermo Fisher Scientific), 2% Normal Armenian Hamster Serum (Innovative Research), 2% Normal Mouse Serum (Thermo Fisher Scientific), and TruStain FcX Plus (Biolegend) in MACS Buffer (PBS + 1% FBS + 1mM EDTA) for 15 minutes on ice. After blocking, samples were incubated with antibodies (Supplemental Table 1) for 30minutes on ice. Stained samples were then fixed in 2% formaldehyde in PBS on ice for 15 minutes.

For intracellular staining, samples were stained for viability and extracellular markers as described above. After staining, cells were fixed with 1x of FOXP3 Fixation/Perm buffer (eBioscience FOXP3/Transcription Factor Staining Buffer Set, Thermo Fisher Scientific) for 30minutes on ice. Following fixation, samples were re-suspended in 1x FOXP3 Perm/Wash buffer for overnight permeabilization at 4 °C. Intracellular antigens were then stained on ice for 30 minutes in 1x perm buffer.

Instrument

LSRII (BD BIosciences)

Software

Collected using FACS Diva software v.9 (BD Biosciences) and analyzed using FlowJo v.10 (Tree Star)

Cell population abundance

Cell population abundance is summarized graphs accompanying flow plots.

Gating strategy

The primary gating strategy is provided in Fig. S1c. Any additional phenotyping performed is also displayed. For example, the gating shown in Fig. 3g is on CD8 TILs defined by the gating strategy shown in Fig. S1c. For antigens with continuous and not discrete expression, positive and negative populations were defined using the florescence minus one (FMO) strategy.

☒ Tick this box to confirm that a figure exemplifying the gating strategy is provided in the Supplementary Information.
